# Functionalized Fluorescent Silica Nanoparticles for Bioimaging of Cancer Cells

**DOI:** 10.3390/s20195590

**Published:** 2020-09-29

**Authors:** Ruth Prieto-Montero, Alberto Katsumiti, Miren Pilare Cajaraville, Iñigo López-Arbeloa, Virginia Martínez-Martínez

**Affiliations:** 1 Departamento de Química Física, Universidad del País Vasco/Euskal Herriko Unibertsitatea (UPV/EHU), 48080 Bilbao, Spain; ruth.prieto@ehu.eus (R.P.-M.); inigo.lopezarbeloa@ehu.eus (I.L.-A.); 2 Departamento de Zoología y Biología Celular Animal, Universidad del País Vasco/Euskal Herriko Unibertsitatea (UPV/EHU), 48080 Bilbao, Spain; alberto.katsumiti@ehu.eus (A.K.); mirenp.cajaraville@ehu.eus (M.P.C.)

**Keywords:** targeting, functionalized fluorescent silica nanoparticles, rhodamine 101, polyethylene glycol, folic acid, HeLa cells

## Abstract

Functionalized fluorescent silica nanoparticles were designed and synthesized to selectively target cancer cells for bioimaging analysis. The synthesis method and characterization of functionalized fluorescent silica nanoparticles (50–60 nm), as well as internalization and subcellular localization in HeLa cells is reported here. The dye, rhodamine 101 (R101) was physically embedded during the sol–gel synthesis. The dye loading was optimized by varying the synthesis conditions (temperature and dye concentration added to the gel) and by the use of different organotriethoxysilanes as a second silica precursor. Additionally, R101, was also covalently bound to the functionalized external surface of the silica nanoparticles. The quantum yields of the dye-doped silica nanoparticles range from 0.25 to 0.50 and demonstrated an enhanced brightness of 230–260 fold respect to the free dye in solution. The shell of the nanoparticles was further decorated with PEG of 2000 Da and folic acid (FA) to ensure good stability in water and to enhance selectivity to cancer cells, respectively. In vitro assays with HeLa cells showed that fluorescent nanoparticles were internalized by cells accumulating exclusively into lysosomes. Quantitative analysis showed a significantly higher accumulation of FA functionalized fluorescent silica nanoparticles compared to nanoparticles without FA, proving that the former may represent good candidates for targeting cancer cells.

## 1. Introduction

Cancer is the second cause of human death worldwide [1,2,3]. Its early diagnosis is the key for an effective treatment to ensure patient survival and cure. Currently, the most used imaging techniques to detect cancer are based on X-ray sources (e.g., computed tomography scan), high magnetic fields (e.g., magnetic resonance imaging), or radioactive substances as tracers (e.g., positron emission tomography scan); however, these techniques can cause side effects in the patients. In fact, to improve the contrast in the images sometimes several scans are required, increasing the chances to suffer side effects [4,5,6,7,8,9]. In the last decade an alternative complementary detection technique, “fluorescence microscopy”, is in expansion since it is less invasive and offers a safe detection with high sensitivity, specificity, and resolution. This versatile technique enables direct imaging of biological structures both in in vitro and in vivo experiments by the use of suitable fluorophores [10,11,12].

Generally speaking, a good fluorophore should fulfill several requirements; (i) high molar extinction coefficients (ε), (ii) high fluorescence quantum yields (Φ) and narrow emission spectra, (iii) low tendency to aggregate, (iv) high Stokes shift in order to prevent reabsorption–reemission processes, and (v) high chemical-, thermal-, and photostability [13]. Many commercial fluorophores meet those conditions but they have some drawbacks to be used as sensors, such as the lack of selectivity for a specific tissue or organelle, low photostability, and poor solubility in physiological media [14].

To overcome these limitations, one strategy is to chemically modify commercial fluorescent dyes to increase their specificity as chemosensors for bioimaging. However, the required multistep chemistry increases the cost of production making these modified dyes an unviable alternative. Another alternative is to associate the fluorescent dyes to a carrier that confers the missing properties of fluorochromes alone. Nanoparticles have been increasingly used as nanocarriers of different molecules because their large surface area serves as platform for the attachment of several molecules. Additionally, nanoparticles help to stabilize hydrophobic components in aqueous media and prevent degradation and inactivation of active compounds [15,16,17,18]. In cancer diagnosis and treatment, targeted nanoparticles can be designed and synthesized to enhance their selective uptake and retention inside tumoral cells [4,19,20,21,22,23,24,25,26,27].

Targeted nanoparticles should be carefully designed in terms of size, structure, composition, and functionalization to balance their stability, diffusion, specificity, and biocompatibility. Currently, there are several nanosystems based on liposomes-, polymeric-, micellar-, metallic-, or protein-based nanoparticles that are approved by the Food and Drug Administration (FDA) for medical applications [4,19,20,21,22,23,24,28,29,30]. Among them, silica nanoparticles (SN), have been receiving special attention due to their wide spectrum of applications. In the last years, SNs have emerged as potential nanocarriers for selective imaging (diagnosis) and targeted drug delivery (therapy) due to their high surface area of easy functionalization, good biocompatibility, optically transparent properties, and low cost [31,32]. Dye-loaded silica nanoparticles have been reported as very promising fluorescent biocompatible nanoplatforms with enhanced photostability and brightness compared to free dyes, thus allowing long-term tracking and higher signal-to-noise ratio fluorescent signals [14,33,34,35,36,37,38,39,40,41,42,43,44,45,46]. Dye molecules can be physically encapsulated or covalently attached to the silica external or internal surface of the nanoparticles [47,48,49,50]. Fluorophores such as rhodamines represent good candidates for labelling nanocarriers because they can be easily associated to silica nanoparticles and show excellent photophysical properties, such as intense absorption and emission bands, in the green-red visible spectra [51].

A part of using fluorescent dyes to label nanoparticles, the external surface of nanoparticles can be further functionalized with molecules that confer stability in aqueous solution. Polyethylene glycol (PEG) is a molecule that improves water stability, minimizes non-specific interactions with other molecules in the extracellular matrix, and does not activate the immune response [26,27,52,53,54,55]. Thus, PEG ensures nanoparticles dispersion and high bioavailability to cells. 

Finally, the selectivity of nanocarriers to cancer cells can be further increased by functionalizing with molecules known to have specific interactions with plasma membrane receptors, which are overexpressed on tumor cells but not on healthy cells. For instance, folate receptors (FRs) exhibit limited expression on healthy cells, but are overexpressed on cancer cells in ovary, mammary gland, colon, lung, prostate, nose, throat, and brain [56,57]. Therefore functionalization of silica nanoparticles with folic acid (FA) turns them into highly selective sensors of cancer cells [43,53,54,57,58,59,60,61,62,63,64,65,66,67].

In this context, in the present work functionalized fluorescent silica nanoparticles were designed and synthesized to target cancer cells. All functionalized silica nanoparticles were (photo)physically characterized (diameter, size distribution, stability, and fluorescent efficiency) in aqueous media. Then, in vitro assays with HeLa cells were used to assess nanoparticles cytotoxicity and, for the most promisor nanoparticles, internalization, and intracellular localization was studied. Finally, internalization of silica nanoparticles with and without FA was quantified in HeLa cells in order to evaluate if functionalization with FA enhances nanoparticles uptake by cancer cells.

## 2. Materials and Methods

### 2.1. Synthesis of the Core-Shell Nanoparticles

All starting materials and reagents were commercially obtained and used without any further modification. Tetraethoxysilane (TEOS) (≥ 99%), ammonium hydroxide solution (NH_4_OH) (≥25% NH_3_ basis), hexadecyltrimethylammonium bromide (CTBA) (≥98%), 3-aminopropyltrimethoxysilane (APTMS) (97%), triethoxymehylsilane (MTES) (≥99%), triethoxyvinylsilane (VTES) (97%), phenyltriethoxysilane (PTES) (98%), triethoxy(octyl)silane (OTES) (98%), rhodamine 101 (R101) (≥99%), N-hydroxysuccimide (NHS) (98%), and N-(3-(dimethylaminopropyl)-N’-ethylcarboiimide (EDC) (≥97%) and folic acid (FA) (≥97%) were purchased from Sigma-Aldrich (Darmstadt, Germany) and polyethylene glycol (PEG) (>95%) from Iris BIOTECH GMBH (Maktredwitz, Germany).

Mesoporous silica nanoparticles (MSNs) were synthesized as it has been described previously [33]. Ormosil nanoparticles (ONPs) were synthesized modifying the MSN synthesis. First, 0.1 g of CTBA was dissolved in 50 mL of NH_4_OH at 60 °C. When CTBA was dissolved, TEOS was added together with a second silica source (MTES or VTES or PTES or OTES) in different ratios from 1:0.1 to 1:1, respectively. After 5 h under vigorous stirring at 60 °C, 0.8 mL of TEOS (1 M in EtOH, 0.8 mmol) and 0.8 mL of an APTMS solution (12% *v/v* in EtOH, 0.007 mmol) was added and kept stirring for 24 h at 60 °C. Then, the temperature was decreased to 25 °C and the mixture was left with vigorous stirring for other 12 h. The NPs were collected by centrifugation at 19,000 rpm at room temperature for 15 min. The collected solid was washed three times with a mixture of Milli Q water/EtOH and a fourth time with EtOH. The surfactant was removed by stirring the NPs with concentrated HCl (0.2 g of HCl in EtOH) for 24 h. The NPs were collected by filtration.

### 2.2. Dye Encapsulation within the NP Core

Rhodamine 101 (R101) dye was directly added to the synthesis gel before silica source addition. The concentration of dye in the synthesis gel and the temperature were varied (5·10^−3^ M–5·10^−4^ M and T = 60–80 °C) to optimize the size of the nanoparticles and the dye loading. When the R101 was completely dissolved in the mixture, the silica source was added; TEOS for MSNs or TEOS and the second silica source for ONPs. After stirring the mixture for 5 h, the shell functionalization with amine groups was carried out as it is explained previously. The corresponding nanoparticles will be denoted MSN-C-R101-**T** and **X**-ONP-R101-**T** (being X the second silica source and T the temperature used during the synthesis).

### 2.3. Grafting of Molecules on NP Surface

R101 and/or folic acid (FA) were grafted to the amine groups of nanoparticles in the external surface by carbodiimide method, following the synthesis described previously [33]. In contrast, silylated-PEG chain (2000 Da) was condensed to the hydroxyl groups in the shell of nanoparticles as it has just been described [33]. The corresponding nanoparticles will be denoted as MSN-S-R101-60, MSN-S-R101-60 –PEG, and MSN-S-R101-60-PEG-FA.

### 2.4. Characterization

The size, shape and morphology of the silica nanoparticles were characterized by electron microscopes, scanning electron microscopy (SEM) and transmission electron microscopy (TEM). SEM images were obtained in a JEOL JSM-6400 (JEOL, Tokyo, Japan) and TEM images were obtained in a Philips SuperTwin CM200 (Thermo Fisher Scientific, Eindhoven, Netherlands) at 200 kV. The nanoparticles size distribution was analyzed by Images-J software (1.52u, National Institute of Health, Bethesda, MD, USA). Dynamic light scattering (DLS) and Zeta potential (Zpot) measurements to analyze the NP size and their stability in suspension were carried out using a Malvern Zetasizer Nano ZS (Malvern Products, Madrid, Spain), which has a Helio-Neon (λ = 633 nm) laser. FTIR spectra were obtained from neat samples in powder using ATR technique in Affinity-1S Shimadzu spectrometer (Izasa Scientific, Barcelona, Spain) (4000–400 cm^−1^ range). The silica nanoparticles absorption spectra were recorded by UV-Vis-NIR Spectroscopy (model Cary 7000, Agilent Technologies, Spain) equipped with two lamps (halogen lamp for Vis-IR region and deuterium lamp for UV region) and an integrating sphere (model Internal DRA 900, Livingston, UK). The fluorescence measurements were recorded with an Edinburgh Instruments Spectrofluorimeter (FLSP920 model, Livingston, UK) equipped with a xenon flash lamp 450 W as the excitation source. The fluorescence spectra were corrected from the wavelength dependence on the detector sensibility. The absolute photoluminescence quantum yields of the dye-containing nanoparticles were measured in an integrated sphere coupled to this spectrofluorimeter. The absorbance at excitation wavelength was obtained by comparing the scatter signal of the dye-loaded hybrid material with a Teflon disk, used as a reference (with a diffuse reflectance of 100%).

The amount of dye uptake into the MSNs or ONPs was estimated photometrically, by dissolving the silica matrix with KOH [33,68,69].

### 2.5. In Vitro Assays

Cells culture: Human cervix adenocarcinoma HeLa cells obtained from ATCC were grown in Dulbecco´s modified Eagle´s medium (DMEM) supplemented with 10% (v/v) fetal bovine serum (FBS) and 50 U/mL penicillin and 50 mg/mL streptomycin, in a humidified 5% CO_2_ cells incubator at 37 °C. For the cell viability assays, cells were grown to monolayer confluency in 96-well microplates. For internalization and subcellular localization studies, cells were seeded in glass-bottom 35 mm petri dishes and subconfluent monolayers were used.

Sample preparation: Samples used for in vitro experiments were prepared by suspending MSN samples directly in PBS buffer (1·10^−4^M). Suspensions were stirred for at least 24 h before the exposures.

Cell viability (MTT) assay: Cytotoxicity of MSN samples (MSN-C-R101-70-PEG and MSN-S-R101-60-PEG) was assessed in HeLa cells using the thiazolyl blue tetrazolium bromide (MTT) assay following manufacturer’s instructions. After exposures, cells were incubated with a 50 mg/mL MTT solution for 3 h at 37 °C. Then, reduced formazan product was extracted from cells with pure DMSO and the absorbance was measured at 570 nm in a Biotek EL 312 microplate spectrophotometer reader (Biotek instruments, Winooski, VT, USA). Cell viability was expressed as the percentage respect to control cells. Differences between control and treated cells were analyzed through the Kruskal–Wallis test followed by Dunn’s post hoc test using the SPSS 23.0 software (IBM, Chicago, CA, USA). Significance level was established at 5% (*p* < 0.05). Four replicates per treatment were performed for all tests and tests were repeated three times each.

Internalization and subcellular localization: To evaluate internalization and subcellular localization of MSN samples through confocal microscopy, cells were incubated for 24 h with 1, 10, and 100 μg/mL of MSN-C-R101-70-PEG and MSN-S-R101-60-PEG-FA in 10% FBS supplemented DMEM culture medium. Unexposed cells were used as control. After exposures, cells were washed three times with culture medium and incubated for 30 min with 50 nM LysoTrackerTM Deep Red (Invitrogen, Paisley, UK) to label cell’s lysosomes, and fixed with 0.4% paraformaldehyde for 10 min at 4 °C. Cells were then washed three times with culture medium and observed under an Olympus Fluorview FV500 confocal microscope (Olympus, Hamburg, Germany). Images were edited using Fiji software (ImageJ 1.49a, National Institutes of Health, Bethesda, MD, USA). To quantify the internalization of MSN samples, cells were incubated for 24 h with 0.1 and 1 μg/mL of MSN samples with and without folic acid (MSN-S-R101-60-PEG-FA and MSN-S-R101-60-PEG, respectively) in 10% FBS supplemented DMEM culture medium. Unexposed cells were used as control. After exposures, cells were washed three times with culture medium and fluorescence of MSN samples was measured at λ_ex_ = 530 nm/λ_em_ = 590 nm in a Cytation 5 Cell Imaging Multi-mode reader (Biotek instruments, Winooski, VT, USA). Fluorescence of MSN samples at 0, 0.0001, 0.001, 0.01, 0.1, and 1 μg/mL was used to normalize internalization data. Differences between control and treated cells were analyzed through Kruskal–Wallis test followed by Dunn’s post hoc test. Differences between the MSN-S-R101-60-PEG-FA and the MSN-S-R101-60-PEG treatments were analyzed through Kruskal–Wallis test followed by Mann–Whitney test. Significance level was established at 5% (*p* < 0.05). All tests were performed using the SPSS software. Four replicates per treatment were performed for all tests and tests were repeated three times each.

## 3. Results and Discussion

**Figure 1:**
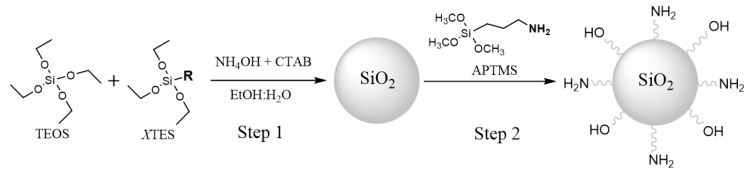
Synthesis of the Ormosil nanoparticles by modified Stöber method with different silica sources (MTES: triethoxymehylsilane, VTES: triethoxyvinylsilane, PTES: phenyltriethoxysilane, and OTES: triethoxy(octyl)silane).

The synthesis of MSNs and ONPs, using the modified Stöber method [70], was directed to obtain silica nanoparticles of around 50 nm, which is considered a suitable size for biomedical applications [52,67,71]. In the case of ONPs different organophilic silica sources (XTES, Figure 1) and ratios respect the main silica source (TEOS), TEOS:XTES 1:0.1, 1:0.5 and 1:1, were studied. The external surface of all MSNs and ONPs was functionalized with amine groups by adding aminopropyltrimethoxysilane (APTMS) after the core nanoparticle formation (Figure 1). 

**Figure 2:**
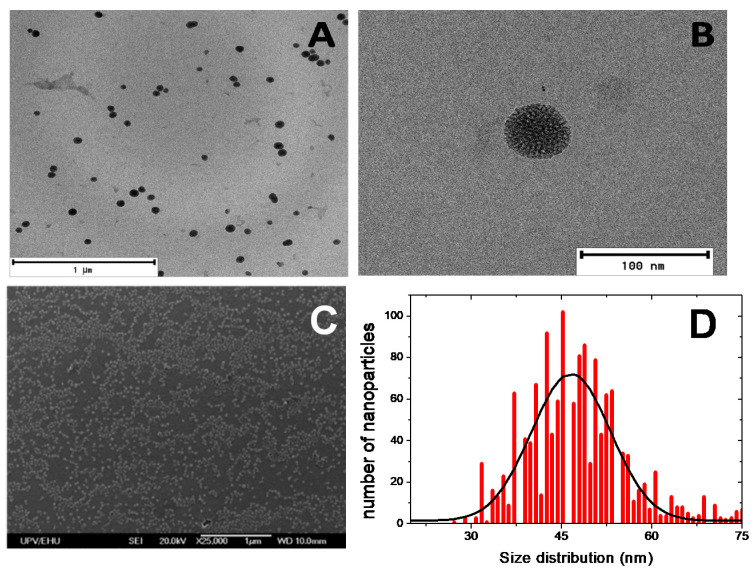
Transmission electron microscopy (TEM) images (**A**,**B**), scanning electron microscopy (SEM) image (**C**) and size distribution (**D**) of mesoporous silica nanoparticles (MSNs).

The MSN size and distribution were analyzed by TEM and SEM (Figure 2 and Figure S1). Electron microscopy images show spherical nanoparticles with a narrow size distribution of 47 ± 10 nm. Regarding the synthesis of ormosil nanoparticles, ONPs, several organophilic silica sources, acting as co-precursors of the silica, at different concentrations, were used with the aim of modulating the hydrophilicity of the porous environment for efficient confinement of the rhodamine fluorescent dye (Table 1). The morphology of these nanoparticles and the size distribution, analyzed by TEM, are depicted in Figure S2 and Table 1. Except for the synthesis with the mixture TEOS:OTES which did not form nanoparticles at any ratio of silica sources, the rest of the samples rendered spherical nanoparticles with a size distribution of around 40–50 nm (Table 1), although in some cases the size distribution is broader than that previously described for MSNs.

Rhodamine 101 (R101), with intense absorption and fluorescence bands (λ_ab_ = 560 nm, ɛ = 8.4·10^4^ M^−1^ cm^−1^, λ_fl_ = 597 nm and Φ = 0.77 in water) was chosen as fluorophore to be loaded into the silica nanoparticles by two different methods: (i) physically embedded within the porous core of MSNs and ONPs and (ii) covalently tethered at their outside surface [25].

In the first approach, to encapsulate R101 within the MSNs, the dye was added to the mixture, before the silica source, at a concentration of 5·10^−4^ M (Table 2). Generally, the dye loaded into silica reached 0.5–1 μmol/g being in the same range as other fluorescent silica nanoparticles, with diameter sizes between 20 and 50 nm, previously optimized with rhodamine 6G [33]. Nevertheless, it is considered low, and with the aim of increasing the dye uptake, R101 was occluded into the different ormosil silica nanoparticles with varied hydrophobic porous environment following the same procedure. As a result, the final dye amount within the ormosil silica nanoparticles was slightly increased (1.5–1.7 fold). However, much higher dye incorporation was found by the rise of temperature of the gel from 60 °C to 70 °C and by augmenting the concentration in the gel to 2.5·10^−3^ M (Table 2). Under these synthesis conditions, particles of around 60 nm diameter and a considerable dye amount embedded were reached with higher dye loaded (>4 μmol/g). Nonetheless, a further increase in the temperature and/or dye concentration in the gel led to a drastic increase in the size of the nanoparticles reaching a diameter of around 500 nm (Table 2 and Figure S3). According to the results obtained in the present study, the sample MSN-C-R101-70 was considered the best fluorescent nanoplatform in this series and was selected for further studies in HeLa cells.

**Figure 3:**
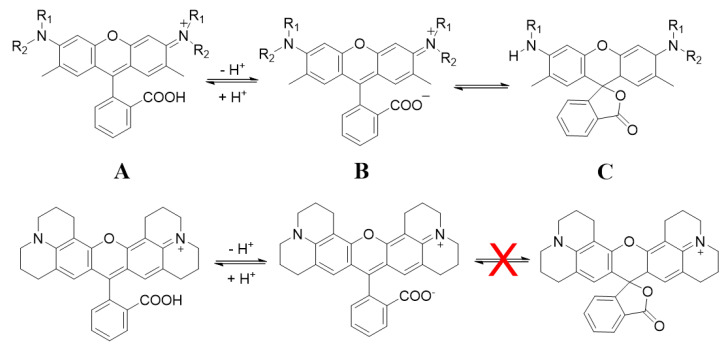
(**top**) General molecular structures of rhodamines in equilibrium and (**bottom**) structure of Rhodamine 101 (R101): cationic (**A**), zwitterionic (**B**), and lactone (**C**) forms.

In the second approach, rhodamine 101 was covalently anchored to the external amine function of MSNs through its carboxylic group by common peptide reaction (sample named as MSN-S-R101-60). Note here that this particular rhodamine, R101, allows this grafting since, after the depronotation process, the formation of spiro-lactone is avoided by the rigidity of the alkyls on N atoms and the zwitterionic form is favored, whereas only lactone species is present in aprotic solvents for the rest of rhodamines and consequently the peptide coupling does not take place (Figure 3) [72,73,74]. The estimated amount of the R101 dye tethered outside, of 22 μmol/g, implied 2-fold increase respect the sample MSN-C-R101-70 with the largest amount of dye occluded inside the core (Table 2). 

Note that the presence of the R101 dye at the external surface in the sample MSN-S-R101-60 should render a more hydrophobic shell and consequently, these nanoparticles were not stable in water and only a relatively stabilized suspension was found for a less polar solvent, such as chloroform. Regarding the sample MSN-C-R101-70, with the dye embedded in the silica core, although the hydrodynamic size in aqueous media, registered by DLS, did not point to a particle aggregation process in water, the low Zpot value indicated poor stability in such media (Table 3). In fact, the photophysical properties of sample MSN-C-R101-70 could not be studied in water due to the particle flocculation while recording the absorption and emission spectra. Thus, it is of crucial importance to improve the stability of these fluorescent nanosystems in aqueous media for their future implementation as bioimaging agents.

To improve the stability in water of samples MSN-C-R101-70 and MSN-S-R101-60, polyethylene glycol (PEG) chains of 2000 Da with a silylated end was anchored to the inherent hydroxyl groups of the external surface of silica nanoparticles (samples denoted as MSN-C-R101-70-PEG and MSN-S-R101-60-PEG in Table 3). The presence of PEG at the silica nanoparticles was checked by FTIR (Figure S4) [26]. After PEGylation of the outside surface of MSN, a drastic increase in the Zpot values (Table 3), from −4 mV up to −23 mV was reached, ensuring good stability in water. 

**Figure 4:**
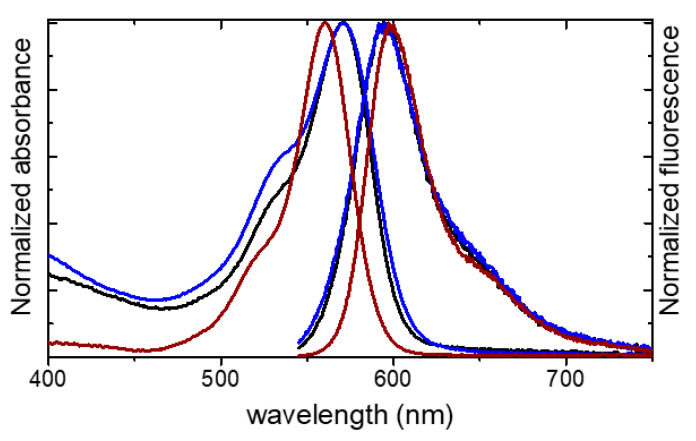
Normalized absorption and emission spectra for MSN-C-R101-70-PEG (black), MSN-S-R101-60-PEG (blue), and rhodamine 101 (brown) in diluted aqueous solution.

The photophysical properties of the PEGylated nanoparticles MSN-C-R101-70-PEG and MSN-S-R101-60-PEG characterized in water are shown in Table 3 and Figure 4. The absorption bands were broader with a more pronounced shoulder, centered at around 525 nm, respect to the R101 in diluted aqueous solution. This could indicate the presence of dye aggregates, which according to exciton splitting bring new absorption bands depending on their geometry. However, as it was previously stated that scattering effects also introduce spectral distortions in the absorption spectra, inducing a “new” shoulder in near position as the current weak vibronic band [75]. Although molecular aggregation cannot be ruled out, the scattering caused by the silica nanoparticles was depicted by the increase of the baseline in the absorption spectra at shorter wavelengths for the samples MSN-C-R101-70-PEG and MSN-S-R101-60-PEG in suspension respect to the dye in solution (Figure 4).

Conversely, the recorded fluorescence spectra for the dye-loaded silica nanoparticles are similar to those obtained for the free dye. The fluorescent nanoparticles render a fluorescence quantum yield of Φ = 0.51 and Φ = 0.25 for MSN-C-R101-70-PEG and MSN-S-R101-60-PEG, respectively (Table 3). The lower fluorescence quantum yield registered in sample MSN-S-R101-60-PEG could be assigned to reabsorption–reemission processes and/or molecular aggregation as a consequence of a higher amount of dye molecules which were distributed mainly at the surface of the nanoparticle.

Although the quantum yield of those fluorescent silica nanoparticles was lower than that of the free dye in water solution (Table 3), the relative brightness of nanoparticles was usually much higher, about tens of times, due to a greater number of fluorophores per particle, enhancing the signal and consequently the sensitivity in the fluorescence imaging detection. Taking into account the area of fluorescence spectra of each suspension and the dye solution, the diameter of nanoparticles, the number of nanoparticles and R101 molecules in the cuvette [76] (see more details in ESI), MSN-C-R101-70-PEG and MSN-S-R101-60-PEG showed a relative brightness of 230 and 260 times respect R101 in water, which made them at least one order of magnitude brighter than the most used quantum dots [41,54,76,77,78]. 

Finally, internalization capacity and cytotoxicity of MSN-C-R101-70-PEG and MSN-S-R101-60-PEG were studied through in vitro experiments in HeLa cells.

**Figure 5:**
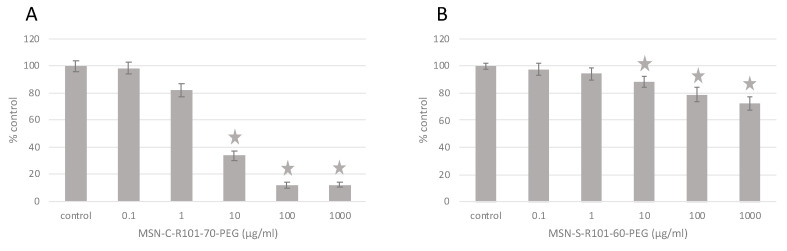
Results of MTT assay for MSN-C-R101-70-PEG (**A**) and MSN-S-R101-60-PEG (**B**). Stars indicate significant differences with respect to controls according to the Kruskal–Wallis test followed by the Dunn’s test (*p* < 0.05).

HeLa cells were exposed to a wide range of concentrations of MSN-C-R101-70-PEG and MSN-S-R101-60-PEG (0.1–1000 μg/mL) and cytotoxicity was assessed through the MTT assay (Figure 5). MSN-C-R101-70-PEG was more cytotoxic than MSN-S-R101-60-PEG, reducing cell viability to less than 40% in HeLa cells exposed to 10 μg/mL, and to less than 20% in those exposed to 100 and 1000 μg/mL. MSN-S-R101-60-PEG reduced cell viability to up to 72.4% in cells exposed to the highest concentration (1000 μg/mL). This difference could be attributed to the possible release of the surfactant CTAB from the core of sample MSN-C-R101-70-PEG, used as the template in the synthesis of the mesoporous nanoparticles, which has been proved to be toxic to many cell types [79,80,81]. In the case of MSN-S-R101-60-PEG the surfactant was previously removed before the R101 grafting, whereas for MSN-C-R101-70-PEG this process cannot be undertaken because it would bleach the R101 dye inside the particles mesopores.

**Figure 6:**
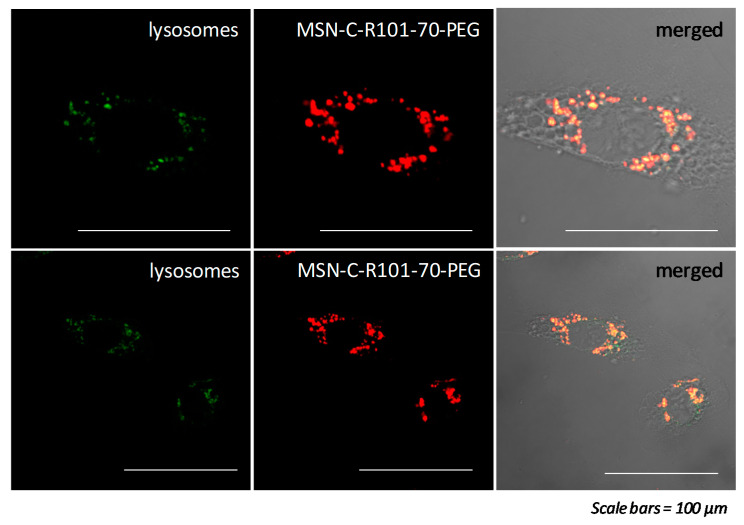
Fluorescence images of MSN-C-R101-70-PEG internalized into lysosomes of HeLa cells. Images show lysosomes (green-**left**), rhodamine 101 from MSN-C-R101-70-PEG (red-**middle**), and merged fluorescence of lysosomes and rhodamine 101 (yellow-**right**). Scale bars 100 μm.

Based on the confocal microscopy analysis, MSN-C-R101-70-PEG were internalized in the cells and specifically accumulated into the lysosomes as shown by the subcellular localization experiments (Figure 6), where the lysotracker (green) and the nanoparticles (red) were co-localized (yellow). The fact that these NPs are localized into lysosomes indicates that they are possibly taken up by endocytosis, as widely reported for silica NPs at similar size range [82,83]. Note that the nanoparticles offer a sharper quality in the bioimaging of lysosomes with respect to commercial lysotrackers. Thus, results indicate that MSN-C-R101-70-PEG could be potentially employed as an alternative lysotracker in cancer cells or other cells that express FR on their plasma membrane. Nevertheless, further studies are needed to confirm it.

As a step forward to enhance the selectivity of the hybrid nanosystem to cervix adenocarcinoma cells, FA was tethered to the shell of MSN-S-R101-60-PEG nanoparticles. The presence of FA at the surface of silica nanoparticles was confirmed by absorption spectroscopy where the characteristic absorption band of FA, centered at 365 nm, was detected together with the main band of R101 at 575 nm, as well as its emission band at 455 nm under 355 nm excitation (Figure S5) [84].

Similar to the results obtained in MSN-C-R101-70-PEG exposures, MSN-S-R101-60-PEG functionalized with FA (MSN-S-R101-60-PEG-FA) were internalized into lysosomes of HeLa cells (Videos 1 and 2, Supplementary Materials). 

The internalization of the nanosystems MSN-S-R101-60-PEG and MSN-S-R101-60-PEG-FA was quantified in HeLa cells exposed to 0.1 and 1 μg/mL of each MSN sample (Table 4). In accordance with previous studies [85], results showed that functionalization with FA significantly increased the internalization of MSNs into HeLa cells, being 13% and 20% higher at 0.1 μg/mL and 1 μg/mL nanoparticle concentrations, respectively (Table 4). 

FA is a manufactured form of folate which is required for the synthesis, repair, and methylation of DNA, as well as for the metabolism of amino acids and RNA [56]. Cancer cells are known to require high levels of folate for cell growth and proliferation; thus, overexpress folic acid receptors on their surface. Folate receptors are a cell surface glycosyl phosphatidylinositol-anchored glycopolypeptides [86], which recognize and internalize FA via endocytosis [87,88]. Folate receptors exhibit limited expression on healthy cells, but are often present in a large number of cancer cells [89]. Thus, as found in the present work, functionalization of nanoparticles with FA increased their uptake by cancer cells.

## 4. Conclusions

Functionalized silica nanoparticles with PEG chains and FA at the external surface, and with rhodamine 101 as fluorescent label embedded into silica nanoparticle’s porous core or covalently linked outside of nanoparticles have demonstrated to be the most suitable fluorescent nanoplatforms for bioimaging of cancer cells. These nanoplatforms showed a suitable dye loading (5–10 mg dye/g nanoparticle), high brightness (230–260 fold increase respect to the dye in solution), improved stability in water (Zpot ~ −23 mV), low cytotoxicity (at concentration ≤ 1 µg/ml), high internalization into HeLa cells and great specificity to cells lysosomes. Functionalization with FA enhanced the internalization of the functionalized silica nanoparticles. These nanosystems offer sharper fluorescence imaging with greater signal-to-noise ratio with respect to commercial lysotrackers, making them promising nanoplatforms for bioimaging of cancer cells.

## Figures and Tables

**Table 1:** Silica nanoparticles synthesized and their average size by TEM.

Name	Silica Source	Size (nm)
MSN	TEOS	47 ± 10
M1-ONP	TEOS:MTES (1:0.1)	44 ± 16
M2-ONP	TEOS:MTES (1:0.5)	38 ± 7
M3-ONP	TEOS:MTES (1:1)	39 ± 18
V1-ONP	TEOS:VTES (1:0.1)	42 ± 7
V2-ONP	TEOS:VTES (1:1)	40 ± 9
P1-ONP	TEOS:PTES (1:0.1)	47 ± 10
P2-ONP	TEOS:PTES (1:1)	49 ± 18
O1-ONP	TEOS:OTES (1:0.1)	-
O2-ONP	TEOS:OTES (1:1)	-

**Table 2:** Synthesis conditions of MSNs and Ormosil Nanoparticles (ONPs): TEOS:XTES ratio, temperature, and initial concentration of R101 in the sol-gel mixture. Average size of nanoparticles (by TEM) and the final amount of loaded dye inside the nanoparticles are given.

Sample	Mixture	Ratio	T (°C)	[Dye]_0_ (M)	Size (nm)	Dye (μmol/g)
MSN-C-R101-60	TEOS	1:0	60	5·10^−4^	47 ± 9	0.56
M-ONP-R101-60	TEOS:MTES	1:1	60	5·10^−4^	54 ± 8	0.81
V-ONP-R101-60	TEOS:VTES	1:1	60	5·10^−4^	29 ± 5	0.96
P-ONP-R101-60	TEOS:PTES	1:1	60	5·10^−4^	39 ± 7	0.94
MSN-C-R101-70	TEOS	1:0	70	2.5·10^−3^	60 ± 9	9.98
M-ONP-R101-70	TEOS:MTES	1:1	70	2.5·10^−3^	58 ± 11	7.54
V-ONP-R101-70	TEOS:VTES	1:1	70	2.5·10^−3^	63 ± 14	4.21
MSN-C-R101-80	TEOS	1:0	80	5·10^−3^	541 ± 73	11.4

**Table 3:** Hydrodynamic diameter (in nm), zeta potential (in mV), absorption peak (λ_ab_ in nm), fluorescence peak (λ_fl_ in nm), and fluorescence quantum yield (Φ_fl_) in water of the dye-loaded silica nanoparticles without and with PEG-coated at their external surface.

Sample	DLS (nm)	Zpot (mV)	λ_ab_	λ_fl_	Φ_fl_	Brightness
MSN-C-R101-70	60	−4.0	-	-	-	-
MSN-C-R101-70-PEG	69	−21.0	572.0	594.0	0.51	230
MSN-S-R101-60-PEG	64	−23.0	571.0	595.0	0.25	260
R101 in water	-	-	560.0	597.0	0.77	1

**Table 4:** Quantification (µg/mL) of MSN-S-R101-60-PEG and MSN-S-R101-60-PEG-FA internalized into HeLa cells after 24 h exposure to 0.1 and 1 μg/mL of each MSN sample (mean ± SD). Different letters indicate significant (*p* < 0.05) differences among groups.

MSN Samples	Control	0.1	1
	**(µg/mL)**	**(µg/mL)**	**(µg/mL)**
MSN-S-R101-60-PEG	0 ± 0.0008 ^a^	0.355 ± 0.029 ^b^	0.491 ± 0.017 ^d^
MSN-S-R101-60-PEG-FA	0 ± 0.0005 ^a^	0.406 ± 0.033 ^c^	0.616 ± 0.023 ^e^

## Supplementary Materials

The following are available online at https://www.mdpi.com/1424-8220/20/19/5590/s1, Figure S1: SEM image of MSNs; Figure S2: TEM images for ORMOSIL nanoparticles using different second silica source and proportions; Figure S3: TEM images for MSN-C-R101-80 sample; Figure S4: FTIR spectra of MSN and MSN-PEG nanoparticles; Figure S5: Normalized absorption and emission spectra for MSN-C-R101-70-PEG, MSN-S-R101-60-PEG, and rhodamine 101 in water, Video 1 and Video 2: Fluorescence images of MSN-S-R101-60-PEG-FA internalized into lysosomes of HeLa cells.
