# Supplementary material for: Functionalized Fluorescent Silica Nanoparticles for Bioimaging of Cancer Cells
Source: Sensors (Basel). 2020 Sep 29;20(19):5590. doi: 10.3390/s20195590 (PMC7582890; doi:10.3390/s20195590)
Supplement: Supplementary file 1 [file sensors-20-05590-s001.pdf]

ESI for:

Functionalized Fluorescent silica nanoparticles for bioimaging of cancer cells

Ruth Prieto-Montero[1], Alberto Katsumiti[2], Miren Cajaraville[2], Iñigo López Arbeloa[1] and Virginia Martinez-Martinez [1*]

[1]Departamento de Química Física, Universidad del País Vasco/Euskal Herriko Unibertsitatea (UPV/EHU) Apartado 644, 48080, Bilbao, Spain.

[2]Departamento de Zoología y Biología Celular Animal, Universidad del País Vasco/Euskal Herriko Unibertsitatea (UPV/EHU) Apartado 644, 48080, Bilbao, Spain.

*Correspondence: virginia,martinez@ehu.eus; Tel.: +34- 946015969

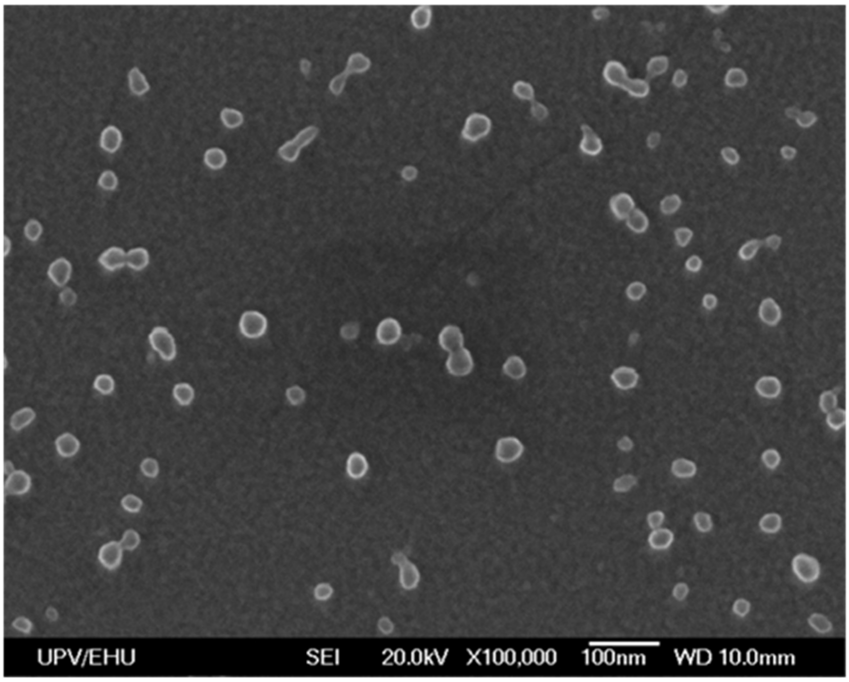

Figure S1. SEM image of MSNs

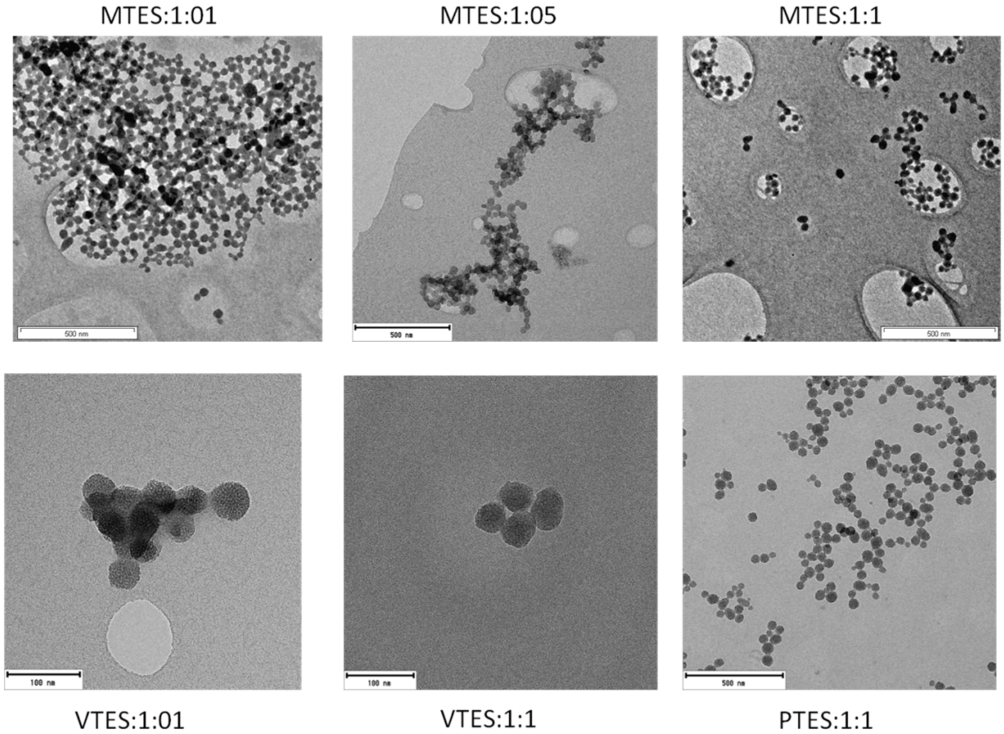

Figure S2. TEM images for ORMOSIL nanoparticles using different second silica source and proportions

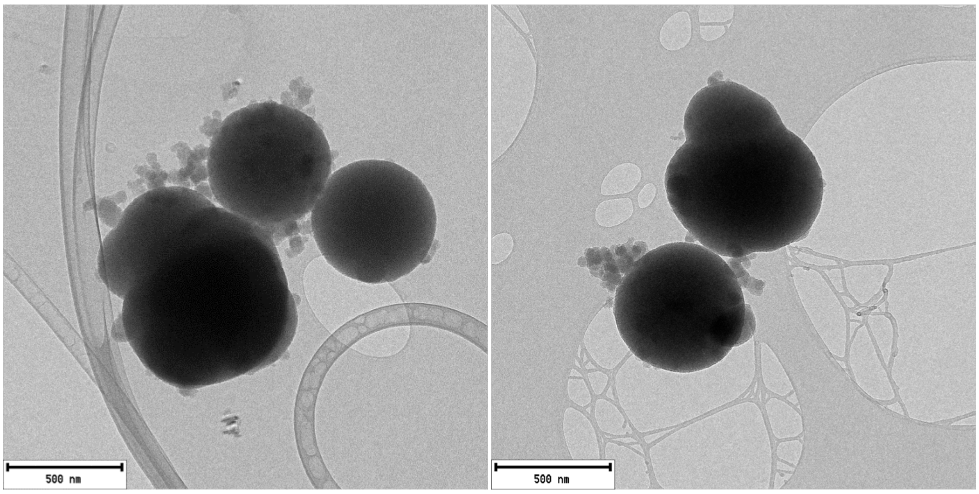

Figure S3. TEM images for MSN-C-R101-80

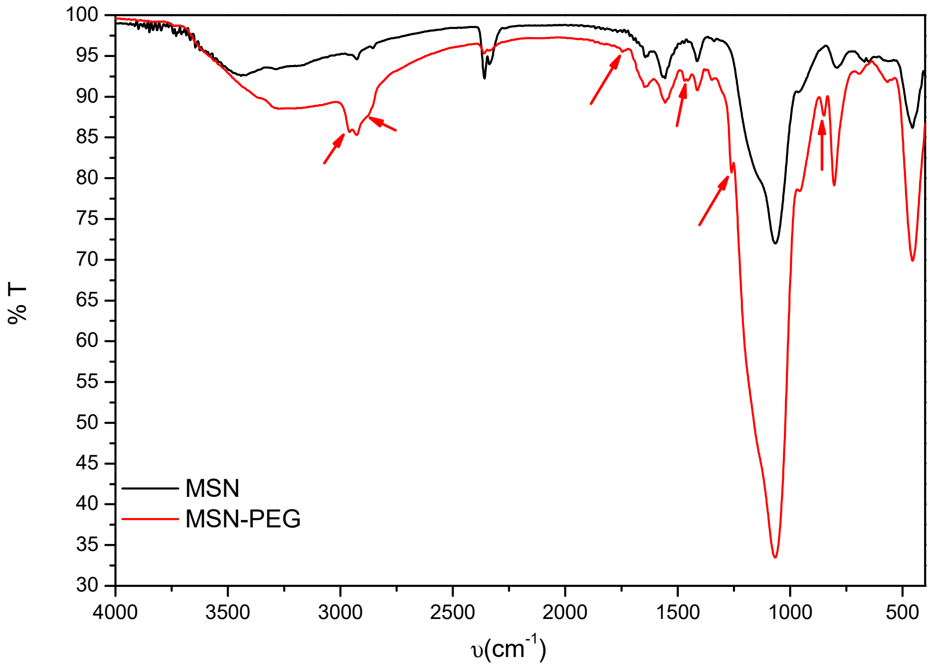

Figura S4: FT-IR spectra of MSN (black) and MSN-PEG (red). Most of the peaks from 400 cm^{-1} to 1200 cm^{-1}, and particularly the main vibration band at 1100 cm−1 are attributed to Si-O-Si vibrations, are present in both samples. The presence of PEG molecules can be verified by the IR band at 2960 cm^{-1} assigned to stretching vibration of CH2 groups of the alkyl chains and the peak at 1460 cm^{-1} to their deformation vibration. The broad band at 2875 cm^{-1} can be assigned to stretching modes of the CH3 groups. The shoulder at 1260 cm^{-1} that appears in the main band may be assigned to stretching vibrations of C-O-C ether bonds.

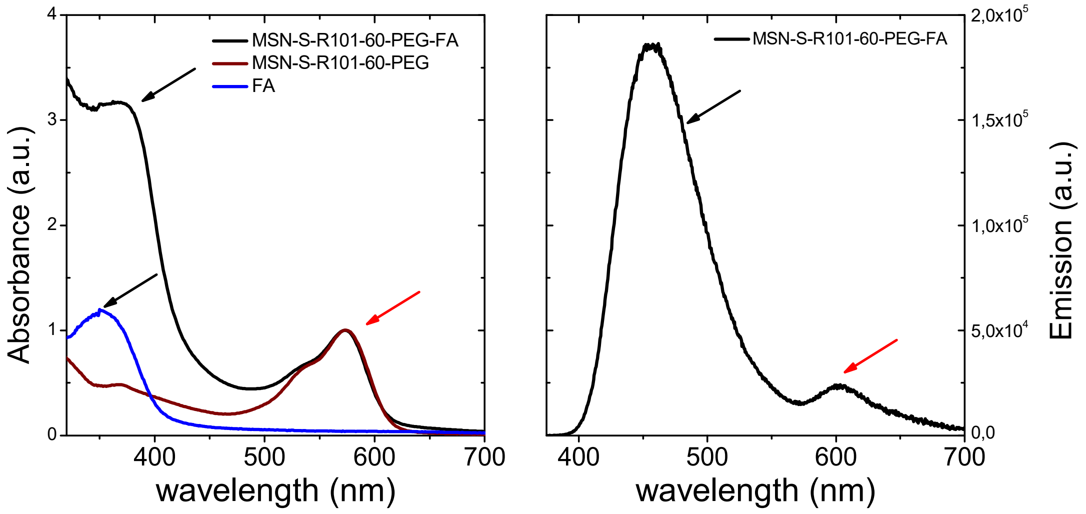

Figure S5. Normalized absorption spectra to the absorption peak of R101 for MSN-S-R101-60-PEG-FA (black), and MSN-S-R101-60-PEG (brown) and FA (blue) (right) and emission spectra for MSN-S-R101-60-PEG-FA under 355 nm excitation wavelength (left). Black and red arrows correspond to absorption (left) and emission (right) bands of folic acid and rhodamine 101, respectively.

Hela-1Rhod101-Lysotracker-02z-63.mov

Video 1. Fluorescence images of MSN-S-R101-60-PEG internalized into lysosomes of HeLa cells; images show lysosomes (green), rhodamine 101 from (red). Scale br 10 µm.

Hela_1Rhod101_Lysotracker_02z_63xZ2-1.avi

Video 2. Fluorescence images of MSN-S-R101-60-PEG internalized into lysosomes of HeLa cells; images show lysosomes (green), rhodamine 101 from (red). Scale br 10 µm.

Technical note:

The relative brightness of the nanoparticles with respect to free dye in solution is calculated following the equation[1]:

Relative Brightness= $(I_{fl}^{NP})\ /\ C^{NP})/(I_{fl}^{R101})\ /\ C^{R101}\ )^{\mathrm{i}}$ [1]

Being I_{fl}^{NP} and I_{fl}^{R101} the fluorescence intensity of the nanoparticles suspension and dye solution, respectively and $C^{NP}$and C^{R101} de number of particles and R101 dye molecules in suspension and diluted solution, respectively

The number of particles is estimated by the concentration of the particles in solution (mg mL^{-1}), the density of the porous silica nanoparticles (1.6 g cm^{-3}) and the average diameter of the nanoparticles (d = 60 nm).

---

[i] Cho, E.B.; Volkov, D.O.; Sokolov, I. Ultrabright fluorescent silica mesoporous silica nanoparticles: Control of particle size and dye loading. *Adv. Funct. Mater.* **2011**, *21*, 3129–3135, doi:10.1002/adfm.201100311.
